# A Rare Case of Takotsubo Cardiomyopathy in a Patient With No Identifiable Emotional or Physical Stressors

**DOI:** 10.7759/cureus.17281

**Published:** 2021-08-18

**Authors:** Swann Tin, William Lim, Anum Humayun, Sean Galligan

**Affiliations:** 1 Internal Medicine, Richmond University Medical Center, Staten Island, USA; 2 Internal Medicine, Richmond University Medical Center, new york, USA; 3 Internal Medicine/Cardiology, Richmond University Medical Center, Staten Island, USA

**Keywords:** takotsubo cardiomyopathy, stressors, chest pain, stress induced cardiomyopathy, left ventricular systolic dysfunction

## Abstract

Takotsubo cardiomyopathy (TCM) is a cardiac condition that presents with features of acute myocardial infarction and transient systolic dysfunction without angiographic findings of obstructive coronary heart disease. Common presenting symptoms include acute substernal chest pain, dyspnea and syncope. It is usually triggered by recent emotional or physical stress such as head trauma, stroke, sepsis, overproduction of catecholamines such as pheochromocytoma or following Myasthenia crisis. We are here to report a case of TCM who does not have any identifiable emotional or physical stress prior to the event. The patient was a 76-year-old Caucasian female with a past medical history of hypertension who presented to the hospital with chest pain which initially was treated as non-ST elevation myocardial infarction (NSTEMI) with aspirin, ticagrelor and heparin infusion. Cardiac catheterization later revealed non-obstructive coronary artery disease but showed akinesis of inferior, apical and anterior walls with hyperdynamic basal segments indicating TCM.

## Introduction

Takotsubo cardiomyopathy (TCM) is a cardiac condition that presents with features of acute myocardial infarction and transient systolic dysfunction without angiographic findings of obstructive coronary heart disease. It is also recognized as broken heart syndrome or stress-induced cardiomyopathy. It usually occurs in elderly individuals following a stressful psychological or physical event.

## Case presentation

A Caucasian female, 76 years, was brought into the emergency room (ER) for a sudden onset mid-sternal chest pain for six days. The pain was intermittent and dull in nature and associated with intermittent palpitations. There were no alleviating or exacerbating factors or any recent stressful events prior to the episode. The patient also has a past medical history of hypertension. She denied any shortness of breath, diaphoresis, dizziness, lightheadedness, dyspnea on exertion, orthopnea, paroxysmal nocturnal dyspnea or extremity edema. She reported being able to walk long distances without feeling short of breath. She denied any family history of cardiac disease or sudden cardiac death. In terms of her social history, she denied any cigarette smoking and illicit drug use but did report social alcohol drinking.

**Figure 1:**
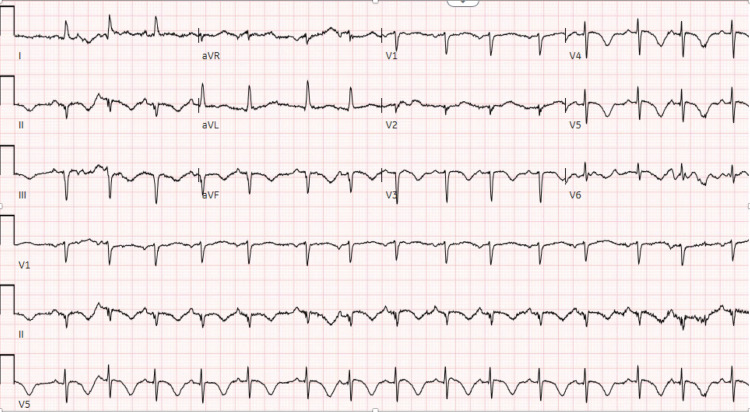
Electrocardiogram (EKG) showing T-wave inversions in inferior leads (lead II, III and aVF) and anterolateral chest leads (V3 to V6)

Upon arrival to ER, her initial vital signs were blood pressure of 164/99 mmHg, heart rate of 70/min, and her oxygen saturation was 98% on room air. Physical examination was essentially normal. Her initial troponin was elevated at 1.1 which further went up to 2.39 in the next three hours. Electrocardiogram (EKG) (Figure 1) showed left axis deviation with prolonged QTc of 564 milliseconds with T-wave inversions in inferior leads (lead II, III and aVF) and anterolateral chest leads (from V3 to V6). Non-ST elevation myocardial infarction (NSTEMI) treatment was initiated in ER and the patient was loaded with aspirin 162 mg, ticagrelor 180 mg and continuous heparin IV infusion.

**Figure 2:**
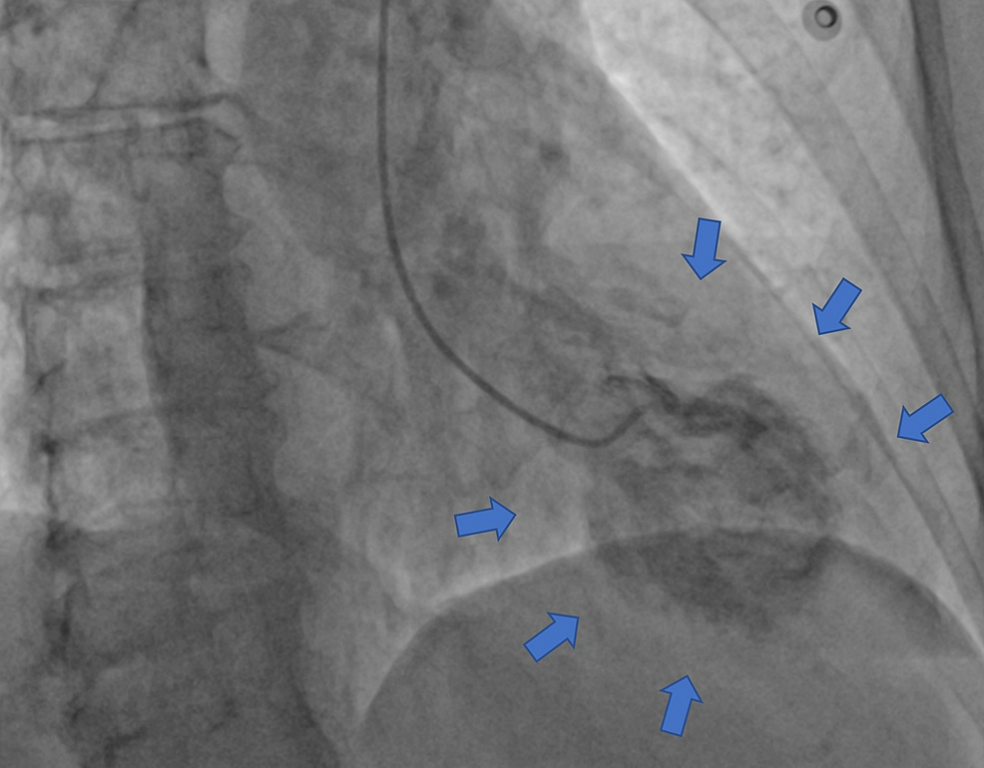
Left ventricular angiogram demonstrating the left ventricle (LV) ballooning (Takotsubo cardiomyopathy or apical ballooning syndrome)

An echocardiogram showed moderate left ventricular systolic dysfunction with ejection fraction (EF) of 30%-35%. She then underwent a cardiac catheterization (Figure 2), which revealed no obstructive coronary artery disease but showed akinesia of inferior, apical and anterior wall with hyper-dynamic basal segments with apical ballooning indicating TCM.

Guideline-directed medical therapy was initiated with metoprolol and lisinopril for low EF (EF <40%). The patient was discharged on the next day and instructed to follow up with the cardiology clinic in 1-2 weeks and to repeat an echocardiogram in 3-6 months.

## Discussion

TCM is an acute cardiac condition that presents with symptoms of acute myocardial infarction and transient left ventricular dysfunction without underlying obstructive coronary artery disease [1-3]. It is commonly seen as apical ballooning of the left ventricle on angiogram which has a close resemblance to a Japanese octopus pot [4]. “Tako” means “octopus” and “tsubo” means “pot.” Current data suggest that it accounts for 2% of patients presenting to the ER with suspected acute coronary syndrome [5]. The incidence is frequently triggered by intense emotional or physical stress [6,7]. Recent studies also noted that the prevalence of TCM following Myasthenia crisis is 15 times higher than the general population [8], and it has increased morbidity and mortality in patients with cannabis use [9]. Our patient here presented with substernal chest pain with elevated troponin levels, which was initially thought to have an acute coronary syndrome. There was no known emotional or physical stress prior, which makes the diagnosis more challenging.

Even though the precise mechanism that causes stress cardiomyopathy is unknown, postulated mechanisms involve diffuse catecholamine-induced microvascular spasm or dysfunction and direct catecholamine-associated myocardial toxicity [7,10]. Common presenting symptoms are acute substernal chest pain, dyspnea and syncope [11]. Common EKG abnormalities found are ST elevation, ST depression, QT interval prolongation, T wave inversion and abnormal Q waves [12]. A transthoracic echocardiogram (TTE) can identify wall-motion abnormalities, especially akinesis or hypokinesis of the apical segment of the left ventricle. The diagnosis can be confirmed with a cardiac angiogram, which reveals no evidence of obstructive coronary vasculature or no signs of plaque rupture [13].

TCM usually improves with conservative management along with the resolution of underlying stressful conditions. Currently, there is no definitive therapy established for this disorder and supportive therapy remains the mainstay of treatment. The 2014 American Heart Association/American College of Cardiology guidelines recommended angiotensin-converting-enzyme inhibitors, beta-blockers, diuretics and aspirin for these patients if they are hemodynamically stable [14]. TCM also has a risk of recurrent attack; however, the appropriate strategy for long-term medical therapy and their effectiveness in preventing recurrence is unclear. For patients with stress cardiomyopathy with intraventricular thrombus, anticoagulation therapy is recommended for a total of three months [15].

TCM is a reversible condition; however, it has considerable rates of death and complications after the acute phase of the disease. According to a recent TCM registry, the rates of death and of stroke/transient ischemic attack are 5.6% and 1.7% per patient-year, respectively [11].

## Conclusions

TCM is a transient medical problem that can mimic an acute coronary syndrome. It has a favorable prognosis and the left ventricular hypokinesis usually improves within a few weeks. However, in some cases, it is associated with serious complications such as hypotension, shock, heart failure, thromboembolism and stroke. Therefore, it is important for physicians to be familiar with various presentations of stress cardiomyopathy and appropriate treatment.
